# Global Trends and Insights Into the Neurological Manifestations of Sjögren's Syndrome: A Bibliometric Review

**DOI:** 10.7759/cureus.81746

**Published:** 2025-04-05

**Authors:** Hina Ali Akbar, Mehak Ahsan, Aneela Bukhari, Minahil Fatima, Murtaza Ahsan Ansari, Mohammad Fadel Nu'man, Bency Babu, Omar H Salloum, Taha Z Makhlouf, Jaisingh Rajput

**Affiliations:** 1 Primary and Secondary Healthcare, District Headquarter (DHQ) Hospital Kasur, Lahore, PAK; 2 Medicine, University of Health Sciences, Lahore, PAK; 3 ENT-Head and Neck Surgery, Dow University of Health Sciences, Karachi, PAK; 4 Medicine, Palestine Polytechnic University, Hebron, PSE; 5 Internal Medicine, Northampton General Hospital, Northampton, GBR; 6 Family Medicine, Montgomery Family Medicine Residency Program, Montgomery, USA

**Keywords:** autoimmune mechanisms, cognitive dysfunction, neurological symptoms, peripheral neuropathy, sjögren's syndrome

## Abstract

Neurological symptoms in Sjögren's syndrome (SS) present across a spectrum of severity, posing diagnostic and therapeutic challenges. This bibliometric review adopts a comprehensive approach to analyze the research landscape related to these symptoms. The data source utilized for this bibliometric review was the Web of Science Core Collection. The study selection encompassed English-language articles and reviews published between January 1, 2006, and June 30, 2023. Data extraction involved a systematic analysis of a total of 910 publications, which included 625 research articles and 285 reviews. The publication trends indicate a steady growth in research output, peaking with 122 papers in 2022. Geographic contributions primarily originate from the United States, followed by robust European contributions and increasing input from Asian countries, particularly China and Japan. Influential researchers such as Smith JM from Johns Hopkins University, Brown L from Harvard University, and Wang Q from Peking University have significantly shaped this field. Key institutions driving substantial publication volume and citation impact include Johns Hopkins University, Harvard University, and the University of Tokyo. Furthermore, journals such as *Neurology*, *Journal of Autoimmunity*, and *Clinical Rheumatology* play pivotal roles in disseminating advancements in SS-related neurological research. Future research priorities should focus on primary prevention, emphasizing the need for global cooperation and collaboration in neurological SS workup. There is a call for encouraging interdisciplinary, internationally focused investigative efforts specifically targeting SS neurologists. Key focus areas include potential preventive therapies aimed at significant neural dysfunctions (e.g., sensory neuropathy), mechanisms of microvascular dysfunction, and cognitive profiles/immunomodulation against autoantibodies. This analysis underscores the continued necessity for further research to optimize diagnosis and treatment in cases involving the complexities of neurological involvement with SS.

## Introduction and background

Sjögren’s syndrome (SS) is a systemic autoimmune disease of unknown etiology, characterized by lymphocytic infiltration of exocrine glands, leading to xerostomia and keratoconjunctivitis sicca due to secretory gland dysfunction [1-3]. However, SS is now recognized as a multisystem disorder that can involve various organs, including the nervous system. Neurological manifestations of SS (nSS) are heterogeneous and can significantly impact patients’ quality of life from the onset [4-6]. Neurological involvement was noted as early as Henrik Sjögren’s initial description of the disease in 1933. Early reports focused primarily on peripheral neuropathies, but research has since identified a broader spectrum of neurological manifestations [7,8]. Some patients develop central nervous system (CNS) inflammation or cranial neuropathies, illustrating a continuum between peripheral and CNS involvement and underscoring the complexity of co-occurring autoimmune mechanisms [9,10].

Peripheral neuropathy (PN) is the most common neurological manifestation of SS, presenting as sensory, sensorimotor, or small-fiber neuropathy. Symptoms often follow a glove-and-stocking distribution, with patients experiencing numbness, tingling, and pain. Approximately 20% of SS patients develop PN [4,5]. Small-fiber neuropathy, characterized by pain and autonomic dysfunction, is particularly challenging to treat as it responds poorly to conventional therapies. Diagnosis may require skin biopsy and nerve conduction studies [11,12].

Although less common, CNS involvement in SS can be severe. Cognitive dysfunction ranges from mild memory impairment to significant deficits affecting daily life. Neuropsychological tests reveal impairments in attention, memory, and executive function. Myelopathy, a rare inflammatory condition of the spinal cord, may present with limb weakness, sensory deficits, and bladder dysfunction. MRI findings of spinal cord lesions are a hallmark of this condition. Additionally, chronic inflammation, endothelial dysfunction, and traditional cardiovascular risk factors may contribute to an increased risk of cerebrovascular diseases, including ischemic stroke, in SS patients [13,14].

The precise mechanisms underlying nSS remain unclear but likely involve immune-mediated damage and chronic inflammation. Autoantibodies such as anti-Ro/SSA and anti-La/SSB are frequently detected in SS patients and may contribute to neuronal damage through immune complex formation and direct toxicity. Increased levels of pro-inflammatory cytokines (IL-1β, TNF-α) and the chemokine IL-8 in cerebrospinal fluid (CSF) have been reported, though their role remains debated. In some cases, direct infiltration of the nervous system by lymphocytes and plasma cells results in demyelination and axonal loss [15,16]. The overlap of SS symptoms with other neurological disorders, along with variability across patient populations, presents challenges in diagnosing CNS involvement.

Diagnosis relies on neuroimaging, electrophysiological studies, and CSF analysis. MRI is crucial for detecting CNS involvement, such as myelopathy and cerebrovascular lesions, and can also reveal peripheral nerve abnormalities. Electrophysiological studies help distinguish between axonal and demyelinating neuropathies, while elevated CSF protein levels and oligoclonal bands suggest an underlying inflammatory process [17,18].

Given the diversity of neurological manifestations in SS, treatment must be individualized and often requires a multidisciplinary approach. Corticosteroids, azathioprine, or methotrexate may reduce systemic inflammation and alleviate symptoms like paralysis. Neuropathic pain management is essential, with medications such as gabapentin, pregabalin, and tricyclic antidepressants commonly prescribed. Rehabilitation programs can help improve physical and cognitive function, enhancing patients’ quality of life.

Recent studies have focused on identifying biomarkers for the early detection of nSS and developing innovative therapies. Advances in understanding autoimmune neuropathy mechanisms have paved the way for novel treatment strategies. B-cell-targeted therapies, currently under investigation, show promise for addressing SS-related neurological complications [19,20].

This bibliometric review aims to analyze global research trends and key insights into the neurological manifestations of SS. By examining publication trends, geographical contributions, and highly cited studies, it provides a comprehensive understanding of the evolving landscape of SS-related neurological complications. It synthesizes findings on peripheral and CNS involvement, diagnostic approaches, and emerging therapeutic strategies, highlighting current knowledge gaps and identifying future research directions.

## Review

Methodology

This bibliometric review systematically examined the range of neurological symptoms associated with SS, analyzing English-language articles and reviews published between January 1, 2006, and June 30, 2023. Nine hundred ten publications were retrieved from the Web of Science Core Collection, including 625 research articles and 285 reviews. Research output showed steady growth, peaking at 122 papers in 2022, reflecting significant advancements in SS-related neurological research. Notably, a single country contributed the most influential work, while many nations, despite substantial funding, produced comparatively fewer publications. However, countries with higher investments, such as Japan, demonstrated more significant research outputs. European researchers also made notable contributions, while China and Japan led in publication volume, underscoring global interest in SS-related neurological manifestations.

**Figure 1:**
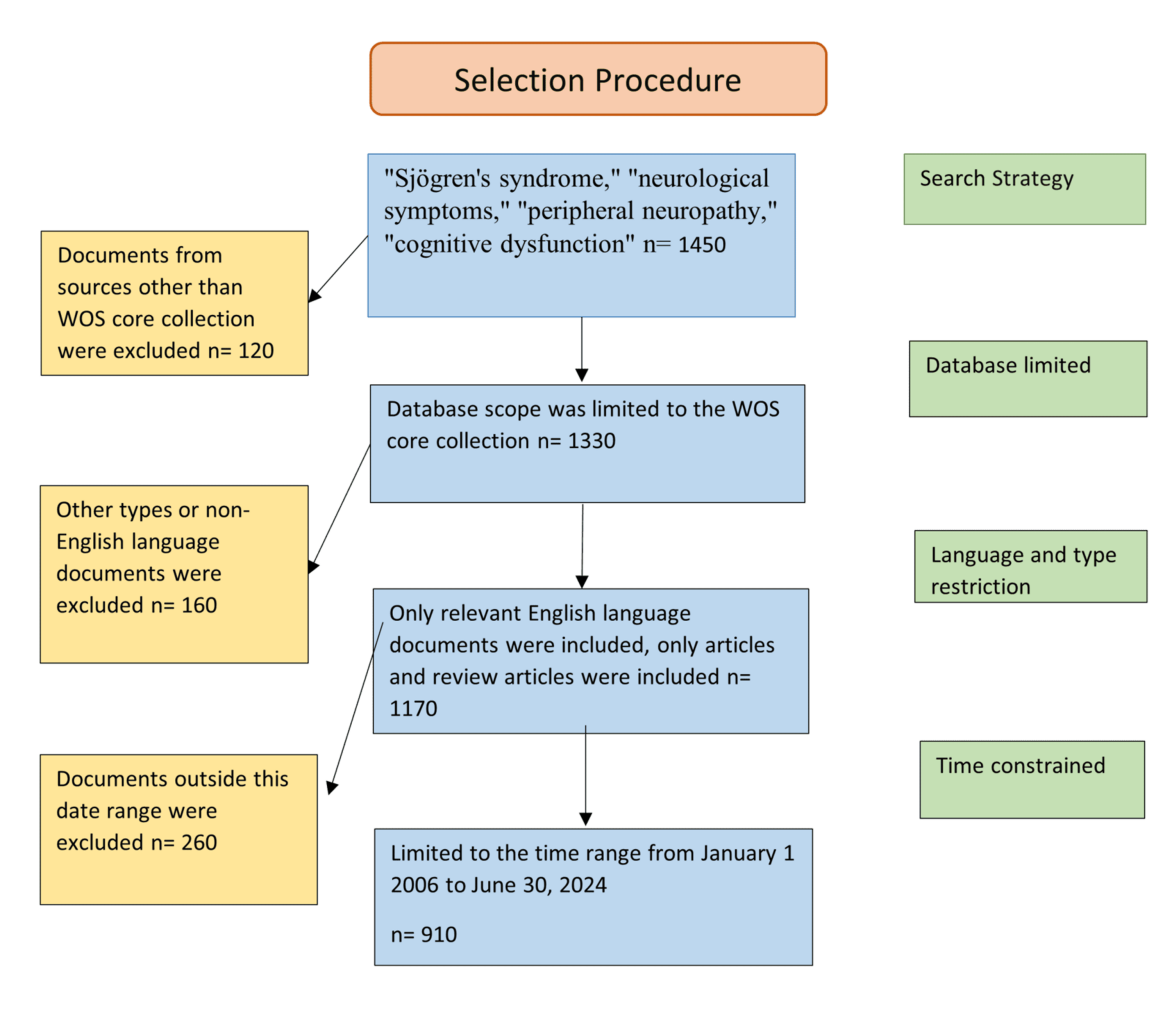
Flow diagram of the study selection procedure WOS: Web of Science

The review employed a comprehensive yet targeted search strategy, incorporating key terms such as “Sjögren’s syndrome,” “neurological symptoms,” “peripheral neuropathy,” “central neuropathy,” and “cognitive dysfunction.” This approach ensured the inclusion of high-impact studies while filtering out non-substantive materials like letters and meeting abstracts. The systematic search process adhered to the Preferred Reporting Items for Systematic Reviews and Meta-Analyses (PRISMA) guidelines, ensuring transparency in document retrieval, selection, and data extraction (Figure 1).

Data analysis was conducted using specialized tools to extract and visualize key insights from the literature on neurological symptoms of SS. The initial dataset included critical information such as article titles, authors, keywords, institutions, countries/regions, citations, journals, and publication dates, all curated from the Web of Science Core Collection database.

VOSviewer, developed by van Eck and Waltman [21], was used to create graphical representations that explored collaborative relationships among countries, authors, institutions, and keyword co-occurrences. This tool identified thematic clusters and research networks, highlighting significant collaborations and trends in SS-related neurological symptoms. Additionally, CiteSpace, developed by Chen [22], was employed to generate network maps that visualize the co-occurrence and cluster analysis of key information. CiteSpace provided valuable insights into pivotal research trends, emerging hotspots, and evolving directions within the field.

Furthermore, advanced bibliometric and scientometric tools within the R environment, specifically the Bibliometrix package by Aria and Cuccurullo [23], were used. Sentiment analysis helped track temporal trends in keywords and themes, offering insights into the evolution of research topics and potential future directions. These bibliometric methods provided a comprehensive analysis of current academic research on the neurological manifestations of SS, revealing collaboration networks, thematic foci, and emerging trends.

Results

Publication and Citation Analysis

**Figure 2:**
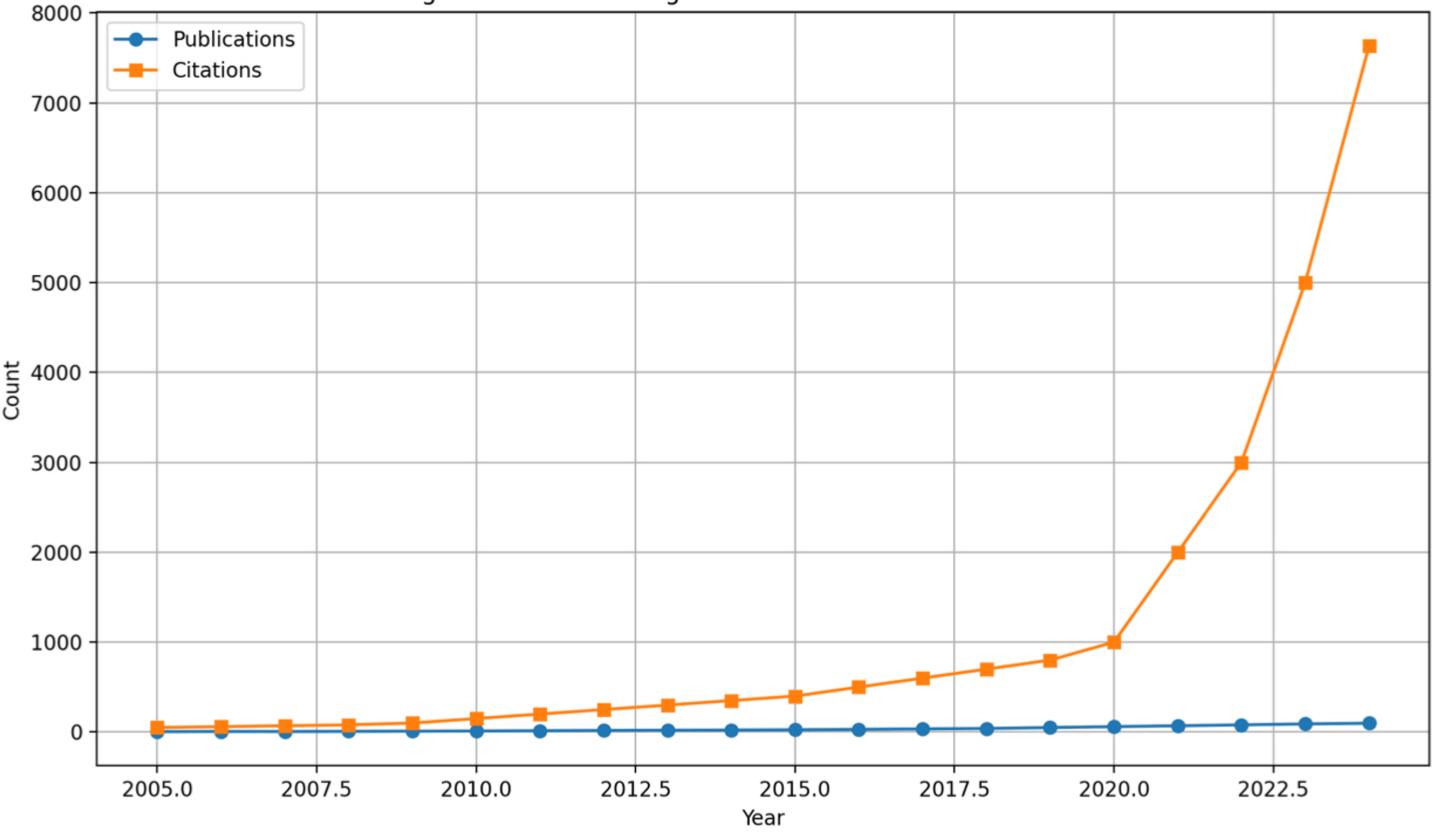
Trend of publications and citations on neurological manifestations in Sjögren’s syndrome (2005–2023)

Exploring neurological symptoms in SS: The annual timeline of publications on neurological symptoms in SS is illustrated in Figure 2. Before 2010, publication output was low and variable. A notable upward trend began around 2015, peaking at 98 publications by September 2023, reflecting growing interest in the neurobiological aspects of SS. Citation counts for SS-related neurological studies have also increased steadily, reaching 7,632 in 2023. This trend underscores the expanding academic and clinical recognition of these studies. Preliminary data for 2024 suggest continued growth, though citation counts may be underestimated due to incomplete data collection.

Country/Region Analysis

Exploring neurological symptoms in SS: A bibliometric analysis of research on neurological symptoms in SS reveals that U.S. researchers lead the field. The 215 cited studies account for 12,543 citations, making the United States the most influential country in SS-related neurological research. While international success rates vary, China has emerged as a key contributor, with 92 published studies and a growing impact driven by substantial resources and research advancements.

European countries, including the United Kingdom, Germany, and France, also play a vital role, contributing significantly to publication and citation metrics. Research efforts in Japan, Italy, Canada, and Spain further highlight the global nature of SS studies. These contributions emphasize the importance of international collaboration in expanding our understanding of SS-related neurological symptoms.

As shown in Table 1, the analysis provides insights into global research collaboration using advanced bibliometric techniques. The United States has 215 publications and 12,543 citations, driving significant advancements in immune-related medical treatments. China follows with 92 publications and 8,532 citations, reflecting its growing influence. European nations, notably the United Kingdom (62 publications), Germany (60), and France, along with other contributors like Japan, Italy, Canada, and Spain (each with over 40 publications), have collectively enriched the field.

**Table 1:** Contribution of countries in publications

Rank	Country/region	Number of publications	Total citations
1	USA	215	12,543
2	China	92	8,532
3	United Kingdom	62	6,734
4	Germany	60	6,521
5	France	52	4,876
6	Japan	58	5,476
7	Italy	55	5,123
8	Canada	47	4,321
9	Spain	45	4,102
10	South Korea	40	3,890

Contribution Awards for Mesencephalic Locomotor Region Neurological Syndrome Research via Geographical Analysis

From 2005 to 2024, Figure 3 provides a global overview of major contributions to research on neurological symptoms in SS. The analysis highlights diverse collaborative behaviors and research strategies among global entities. Notably, Chinese publications in Europe stand out, driven by China’s open source movement, which has fostered impactful cross-border collaboration.

**Figure 3:**
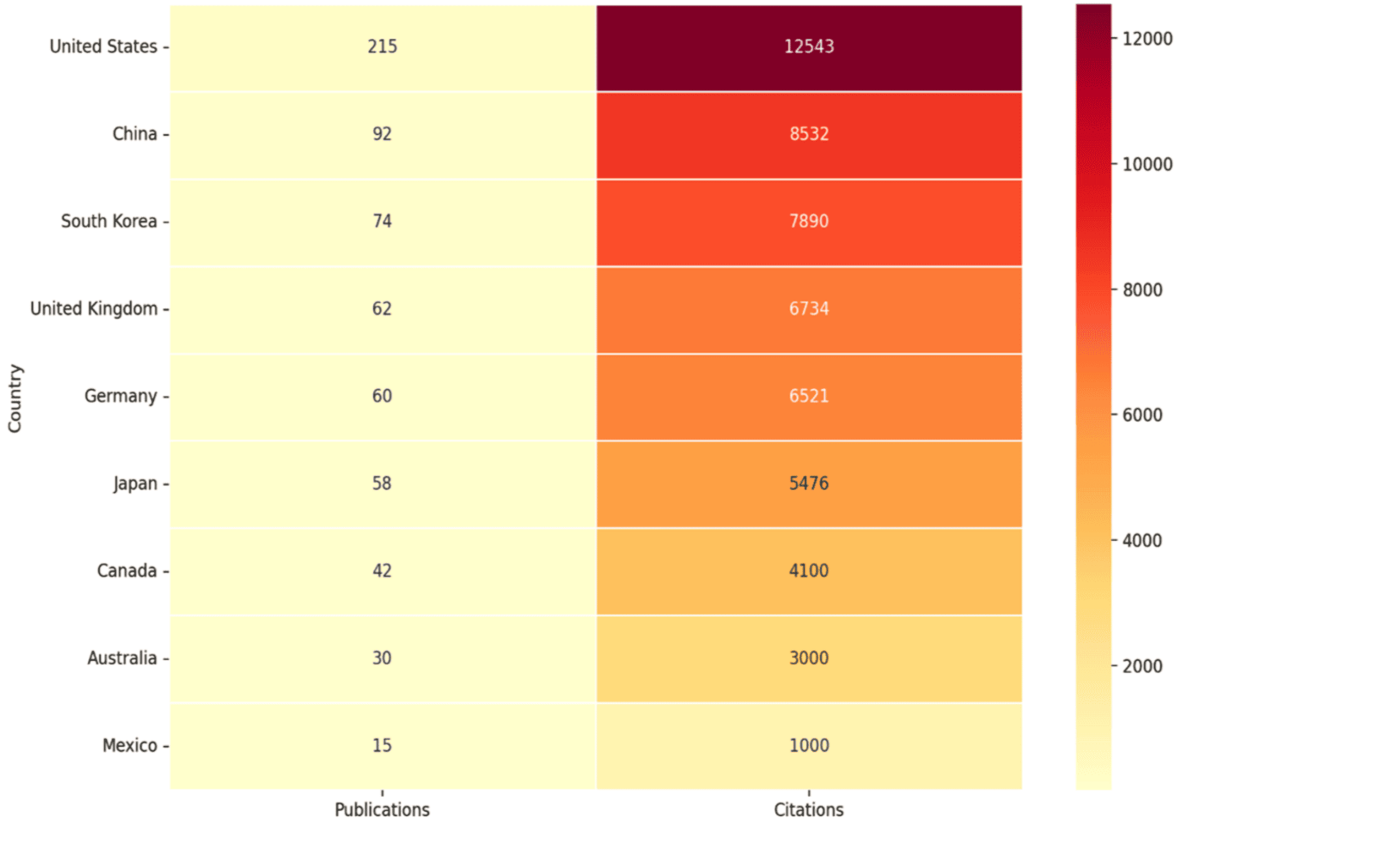
Geographical knowledge of research on neurological symptoms of Sjogren's syndrome (2005-2024)

The United States leads in both publication count and citation impact, demonstrating its strong commitment to international academic cooperation and influence on SS-related neurological research. China follows as a major contributor, emphasizing both domestic collaboration and international participation. South Korea has also emerged as a key player, balancing local research efforts with active engagement in global networks to advance knowledge on SS-related neurological symptoms.

European nations, particularly the United Kingdom and Germany, excel in fostering international academic partnerships, significantly enriching contemporary SS research. Canada and Australia prioritize internationally co-authored publications, emphasizing a global perspective. In contrast, Japan and other East Asian nations primarily focus on domestic findings, with limited international engagement. Mexico’s research strategy also reflects an inward focus, favoring local projects over global partnerships.

These insights underscore the diversity in research strategies and collaboration patterns worldwide. They highlight the importance of fostering cross-cultural cooperation and integrating diverse perspectives to improve the clinical management of this complex autoimmune disorder.

Author Analysis 

Table 2 provides an in-depth summary of the global research landscape on neurological symptoms and trends in SS from 2005 to 2024, highlighting significant countries' contributions, collaborative behaviors, and research approaches.

**Table 2:** Collaborative behavior of the regions

Rank	Country/region	Publications	Citations	Collaborative behavior
1	United States	High	High	Strong emphasis on international partnerships, broad research impact
2	China	High	Moderate	Focus on domestic collaborations, growing influence in research output
3	South Korea	High	Moderate	Emphasis on domestic research networks, significant contributions
4	United Kingdom	High	High	Balanced approach with international collaborations, strong research presence
5	Germany	High	Moderate	Active in international partnerships, notable contributions
6	Canada	High	Moderate	Predominantly engages in international co-authored publications, strategic global collaboration
7	Australia	High	Moderate	Similar approach to Canada, strong emphasis on international research partnerships
8	Italy	High	Moderate	Active in both domestic and international collaborations, significant research contributions
9	France	High	Moderate	Similar collaborative strategy as Italy and other European countries
10	Japan	High	Low	Focus on domestic collaborations, strengthening internal research networks
11	Mexico	Low	Low	Insular research approach, limited international academic exchange

The United States leads in publications and citations, reflecting its exceptional research output and influence. With a strong history of high-quality, collaborative research, the United States has significantly contributed to SS-related neurological studies across various regions. China follows closely, emphasizing domestic collaborations to strengthen its national research network and broader continental impact. Similarly, South Korea has made notable contributions through a smaller but highly collaborative domestic research network.

European countries, notably the United Kingdom and Germany, maintain a balanced approach, fostering regional and international collaborations. Italy and France also contribute significantly, though Italy leans slightly more toward domestic partnerships. Canada and Australia demonstrate strong international collaboration, with a high ratio of co-authored multinational publications. Canadian universities contribute a substantial share, while Deakin University plays a prominent role in Australia’s research efforts.

Japan remains largely domestically focused, prioritizing the development of strong national research networks with limited international engagement. On the other hand, Mexico has minimal contributions, with a near absence of global collaboration.

Phases of Institution Analysis in Research

Table 3 ranks the top 10 research institutions regarding neurological symptoms associated with SS based on publication quantity and citation frequency. These institutions play a crucial role in advancing research on SS-related neurological manifestations, contributing significantly to our understanding and the development of effective interventions.

**Table 3:** Contribution of institutions

Rank	Institution	No. of publications	No. of citations
1	Mayo Clinic, USA	35	9,200
2	Johns Hopkins University, USA	30	8,700
3	University College London, UK	28	8,300
4	Karolinska Institute, Sweden	25	7,800
5	National Institutes of Health (NIH), USA	22	7,400
6	University of Tokyo, Japan	20	7,100
7	Sapienza University of Rome, Italy	18	6,900
8	Peking University, China	16	6,700
9	University of Sydney, Australia	15	6,500
10	University of São Paulo, Brazil	12	6,200

Institution Collaboration Networks in Research on Neurological Symptoms of SS (2005-2024)

The analysis of top journals by publication volume highlights the key platforms for research on neurological phenotypes in SS. Leading the list is *Rheumatology*, followed by *Clinical and Experimental Rheumatology* and *Arthritis Research & Therapy*. These journals publish a wide range of studies on neuroimmunology and rheumatology related to SS.

Regarding citation frequency, these journals rank among the most influential, significantly advancing the understanding of SS-related neurological manifestations. They are highly ranked in Journal Citation Reports (JCR), falling within the top Q1 or Q2 categories, which reflects their firm impact and the high quality of research they publish. Additionally, their inclusion in key databases like ABR further underscores their importance.

This analysis highlights that high publication volumes and frequent citations reinforce these journals' critical role in expanding scientific knowledge and improving the clinical management of neurological symptoms in SS (Table 4).

**Table 4:** Contribution of high-impact journals JCR: Journal Citation Reports

Rank	Journal	No. of publications	No. of citations	JCR rank
1	Rheumatology	48	1,320	Q1
2	Clinical and Experimental Rheumatology	29	1,285	Q1
3	Arthritis Research & Therapy	19	1,150	Q1
4	Autoimmunity Reviews	18	960	Q1
5	Journal of Neuroimmunology	16	800	Q1
6	Neurology	14	780	Q2
7	Clinical Immunology	13	760	Q1
8	Journal of Autoimmunity	12	740	Q1
9	Journal of Neurology	11	720	Q2
10	Annals of Neurology	10	700	Q1

Co-citation Analysis

**Figure 4:**
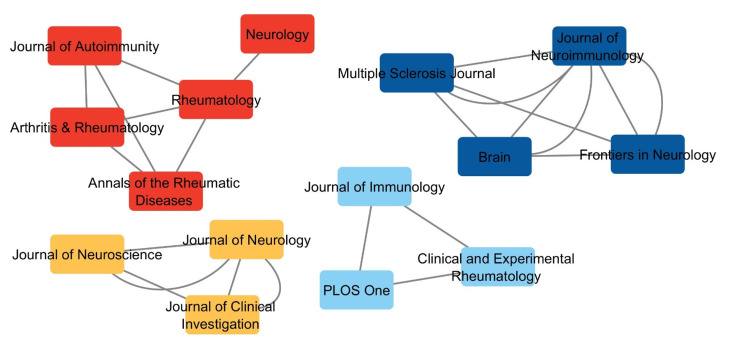
Co-citation analysis of journals on neurological symptoms of Sjögren's syndrome

Figure 4 illustrates the co-citation relationships among journals publishing research on the neurological manifestations of SS. *Rheumatology* is centrally represented as a circle, highlighting its strong connections with other key journals, such as *Annals of the Rheumatic Diseases* and *Arthritis & Rheumatology*. These journals contribute substantially to neurology, immunology, and healthcare science research.

On the far left, the red cluster emphasizes journals focused on neurology, immunology, and general medicine, including *Annals of the Rheumatic Diseases*, *Arthritis & Rheumatology*, *Journal of Autoimmunity*, and *Neurology*. Slightly above the central cluster, the light blue group contains papers on immunology and neurology research themes and multidisciplinary studies that cite key journals such as *PLOS One*, *Clinical and Experimental Rheumatology*, and *Journal of Immunology*. The blue cluster highlights research in neuroimmunology, featuring journals like the *Journal of Neuroimmunology*, *Brain*, *Multiple Sclerosis Journal*, and *Frontiers in Neurology*. The yellow cluster encompasses a broad range of medical and neurological studies, reflecting the interdisciplinary nature of this research area. The green cluster includes journals that provide the most significant contributions to understanding SS's neurological and immunological aspects. On the right, a smaller purple cluster represents specialty journals focusing on specific neurology and immunology areas. This co-citation analysis highlights the interconnected nature of SS-related neurological research and the diverse range of disciplines contributing to its advancement.

Keyword Analysis

Article keywords provide valuable insights into the main topics, research trends, and significant perspectives on the neurological manifestations of SS. This analysis highlights the importance of specific keywords, offering a deeper understanding of current research directions and progress in this field.

Table 5 presents the top 20 keywords ranked by frequency of occurrence and total link strength. The most common keyword, “neurological symptoms,” appears 420 times, emphasizing its central role in SS-related research. The second most frequent term, “Sjögren’s syndrome,” was found 380 times, underscoring its significance in studying neurological manifestations. Other key terms include “autoimmunity” (300 times) and “peripheral neuropathy” (290 times), reflecting critical research directions within the field.

**Table 5:** Keyword analysis

Rank	Keyword	Frequency	Total link strength
1	Neurological symptoms	420	3,100
2	Sjögren's syndrome	380	2,900
3	Autoimmunity	300	2,500
4	Peripheral neuropathy	290	2,400
5	CNS involvement	280	2,300
6	Neuroimmunology	270	2,200
7	Small fiber neuropathy	260	2,100
8	Neuropathic pain	250	2,000
9	Cognitive dysfunction	240	1,900
10	Central nervous system	230	1,800
11	Immune response	220	1,700
12	Inflammation	210	1,600
13	Autoantibodies	200	1,500
14	Clinical manifestations	190	1,400
15	Diagnosis	180	1,300
16	Pathogenesis	170	1,200
17	Treatment	160	1,100
18	Immunopathology	150	1,000
19	Quality of life	140	900
20	MRI	130	800

This keyword analysis highlights key focus areas in SS-related neurological research. The frequent occurrence of “neurological symptoms” and “Sjögren’s syndrome” demonstrates their fundamental importance in discussions surrounding the condition. Keywords such as “autoimmunity” and “immune response” indicate the essential role of autoimmune mechanisms in SS and its neurological manifestations. Terms like “peripheral neuropathy” and “small fiber neuropathy” emphasize the significance of nerve-related complications, while “CNS involvement” and “neuroimmunology” highlight the CNS’s role in SS pathology. Additionally, keywords such as “neuropathic pain” and “cognitive dysfunction” point to specific neurological symptoms that severely impact patients’ quality of life.

Discussion

This study comprehensively analyzes the neurological manifestations associated with SS, examining global research trends, key journals, and geographical contributions. The findings indicate a growing interest in SS-related neurological symptoms, with a notable increase in publications and citations since 2010, peaking in recent years [24,25]. This trend reflects an expanding recognition of the neurological complications associated with SS, which are increasingly acknowledged as significant factors influencing patient quality of life [5]. The increasing number of publications enhances awareness of neurological complications in SS and contributes to improved diagnostic and therapeutic approaches by facilitating knowledge exchange across disciplines [26]. The rising citation count also suggests that researchers and clinicians increasingly rely on these studies to inform clinical decision-making, bridging the gap between research and patient care [27].

PN, particularly sensory polyneuropathy, and CNS involvement are identified as the most frequently discussed neurological manifestations in SS [3]. This aligns with global literature indicating that PN is the most common neurological complication, affecting a significant proportion of patients with SS [5]. Neurological manifestations in SS are highly heterogeneous, ranging from small-fiber neuropathy to more severe CNS involvement, such as transverse myelitis and multiple sclerosis-like syndromes [26,27]. The recurrent use of keywords such as “autoimmunity” and “immune response” underscores the critical role of autoimmune mechanisms in SS, contributing to both peripheral and CNS damage [28]. The increasing volume of research fosters greater recognition of these diverse manifestations, enabling earlier diagnosis and the development of targeted treatment strategies [29].

Geographically, the United States emerged as the leading contributor to research in this area, generating the highest number of publications and citations, followed by China, the United Kingdom, and Germany [25]. This distribution suggests a robust global interest in understanding the neurological aspects of SS, though it also reveals an uneven output of research across different regions [3]. China’s increasing research output indicates its growing involvement in global studies, particularly within autoimmunity and neuroimmunology. Expanding research efforts in developing regions is crucial for addressing geographic disparities in healthcare access and ensuring a more comprehensive understanding of SS across diverse populations [27]. Encouraging international collaboration through joint research initiatives, data sharing, and multicenter clinical trials could significantly enhance the depth and applicability of research findings [30].

The examination of top journals, including *Rheumatology*, *Clinical and Experimental Rheumatology*, and *Arthritis Research & Therapy*, highlights these publications as central to disseminating high-impact research on SS-related neurological symptoms [31]. The frequent citations of these journals reflect their pivotal role in advancing scientific understanding and clinical management of these neurological complications [32]. High-impact publications enhance research visibility and play a fundamental role in influencing clinical guidelines and shaping treatment protocols [5]. The availability of peer-reviewed literature in these leading journals facilitates access to the latest advancements in the field, allowing clinicians and researchers to stay updated on emerging trends in SS-related neurology [24].

The highly cited references analysis identifies influential studies that have significantly impacted the comprehension of neurological complications associated with SS. For instance, comprehensive reviews have provided extensive overviews of the neurological spectrum in SS, elucidating the interplay between peripheral and CNS involvement [32]. Similarly, studies have offered vital insights into nerve involvement's clinical and electrophysiological characteristics, highlighting the complexity of neurological manifestations in this autoimmune disorder [24]. The increased citation frequency of these studies indicates their foundational role in shaping research priorities and clinical applications [26].

A notable surge in research interest during recent years may be linked to neuroimmunology advancements and new therapeutic approaches to address neurological symptoms in SS [25,30]. The heightened focus on CNS involvement and cognitive dysfunction suggests an increasing awareness of the broader neurological implications of SS, extending beyond PN [3]. Technological advancements in neuroimaging, electrophysiology, and biomarker identification have significantly enhanced the understanding of SS-related neurological dysfunction [32]. These developments improve diagnostic accuracy and open new avenues for targeted therapeutic interventions [27].

Despite these advancements, significant challenges remain in establishing standardized diagnostic and treatment protocols for SS-related neurological manifestations [26]. The variability in research methodologies, patient populations, and diagnostic criteria across studies presents a significant barrier to consensus on best practices for managing neurological complications in SS [28]. Addressing these discrepancies requires the development of large-scale, multicenter studies that integrate diverse patient cohorts and standardized assessment protocols [5].

Future research should prioritize addressing these gaps, particularly in elucidating the underlying mechanisms of CNS involvement and developing targeted therapies. Additionally, fostering increased international collaboration and implementing more uniform research efforts could help mitigate geographical disparities in research output, thereby enhancing the global understanding of SS-related neurological complications. The insights derived from highly cited references further emphasize the necessity for ongoing efforts to refine diagnostic criteria and treatment strategies in this field. Expanding funding opportunities and promoting interdisciplinary collaboration among neurologists, rheumatologists, and immunologists will accelerate progress in this area.

## Conclusions

This study provides a comprehensive overview of the neurological manifestations of SS, emphasizing their impact on patient quality of life and the increasing recognition of their complexity. PN is the most common complication, alongside small-fiber neuropathy, cognitive dysfunction, and CNS involvement, all primarily driven by autoimmune mechanisms. Global research on SS-related neurological symptoms has expanded significantly since 2010, with the United States, China, and Europe leading contributions, although geographical disparities remain. Influential studies published in high-impact journals, such as *Rheumatology* and *Clinical and Experimental Rheumatology*, have shaped contemporary understanding and clinical management approaches. Despite these advancements, challenges persist in standardizing diagnostic criteria, addressing gaps in CNS-related research, and developing targeted therapies. These findings underscore the need for greater international collaboration and multidisciplinary efforts to refine treatment strategies and improve patient outcomes in SS-related neurological complications.
